# Practical Lessons in Nursing

**Published:** 1887-08

**Authors:** 


PRACTICAL LESSONS IN NURSING. THE NURSING AND CARE OF THE NERVOUS AND INSANE. By Charles K. Mills, M. D., Professor of Diseases of the Mind and Nervous System in the Philadelphia Polyclinic and College for Graduates in Medicine; Neurologist to the Philadelphia Hospital; Lecturer on Mental Diseases in the University of Pennsylvania; Consulting Physician to the Insane Department of the Philadelphia Hospital, etc. Philadelphia: J. B. Lippincott Company. London: 10 Henrietta street, Covent Garden. 1887.

With the recognition that a good nurse will always be loyal to the doctor, and thus render the best service to the patient, it is evident that such teaching as is found in this book should be appreciated by the medical profession.

